# The p53 endoplasmic reticulum stress-response pathway evolved in humans but not in mice via PERK-regulated *p53* mRNA structures

**DOI:** 10.1038/s41418-023-01127-y

**Published:** 2023-02-22

**Authors:** Leila Fusée, Norman Salomao, Anand Ponnuswamy, Lixiao Wang, Ignacio López, Sa Chen, Xiaolian Gu, Stavros Polyzoidis, Sivakumar Vadivel Gnanasundram, Robin Fahraeus

**Affiliations:** 1 Inserm U1131, 27 Rue Juliette Dodu, 75010 Paris, France; 2 Department of Medical Biosciences, Umea University, 90185 Umea, Sweden; 3 Biochemistry-Molecular Biology, Faculty of Science, Universidad de la República, Iguá 4225, 11400 Montevideo, Uruguay; 4 Department of Neurosurgery, AHEPA Hospital, Aristotle University of Thessaloniki, Thessaloniki, Greece; 5 RECAMO, Masaryk Memorial Cancer Institute, Zluty kopec 7, 65653 Brno, Czech Republic

**Keywords:** RNA, Tumour-suppressor proteins, Kinases

## Abstract

Cellular stress conditions activate p53-dependent pathways to counteract the inflicted damage. To achieve the required functional diversity, p53 is subjected to numerous post-translational modifications and the expression of isoforms. Little is yet known how p53 has evolved to respond to different stress pathways. The p53 isoform p53/47 (p47 or ΔNp53) is linked to aging and neural degeneration and is expressed in human cells via an alternative cap-independent translation initiation from the 2nd in-frame AUG at codon 40 (+118) during endoplasmic reticulum (ER) stress. Despite an AUG codon in the same location, the mouse *p53* mRNA does not express the corresponding isoform in either human or mouse-derived cells. High-throughput in-cell RNA structure probing shows that p47 expression is attributed to PERK kinase-dependent structural alterations in the human *p53* mRNA, independently of eIF2α. These structural changes do not take place in murine *p53* mRNA. Surprisingly, PERK response elements required for the p47 expression are located downstream of the 2nd AUG. The data show that the human *p53* mRNA has evolved to respond to PERK-mediated regulation of mRNA structures in order to control p47 expression. The findings highlight how *p53* mRNA co-evolved with the function of the encoded protein to specify p53-activities under different cellular conditions.

## Introduction

The activation of p53 in response to changes in cellular conditions helps coordinate stress signaling pathways by altering downstream gene expression in order to deliver cell biological responses that are suitable to counteract the inflicting damage [1, 2]. To diversify its functional activity, p53 is subject to numerous post-translational modifications and protein-protein interactions that together control pathways governing cell cycle arrest, DNA repair, metabolism, or induce irreversible changes such as senescence or apoptosis [3]. The expression of isoforms lacking certain domains of p53 forming homo- or hetero-oligomers, further expands the p53 activity repertoire [4, 5]. The human p53 isoform, p53/47 (p47 or p53NΔ40) is derived from alternative cap-independent initiation of translation following stress to the endoplasmic reticulum and lacks the first 40 amino acids, including the first transactivation domain (TA1) and the MDM2-binding site [5]. It retains the oligomerization domain and can form homo-or hetero-oligomers with p53, thereby altering the functional activity of p53 [6–8]. The equivalent of human p47 in murine cells, p44, was discovered following retrovirus infection of a murine cell line [9]. Overexpression of p44 in mice with a p53-WT background resulted in a progeroid phenotype with severe pre-mature aging and altered stem cell pluripotency that was not observed in a p53-null background, suggesting that the capacity of p44 to regulate cellular pathways is dependent on the expression ratio between the full-length p53 and the p44 isoform [10, 11]. A link between neurodegenerative disease and p44 was reported via the regulation of tau kinases. Furthermore, p47 was detected in regenerative processes in neural progenitor cells and gliosis, and elevated p47 expression was observed in xenografts derived from glioblastoma multiforme (GBM) [12–14].

The endoplasmic reticulum (ER) is the major cellular organelle involved in protein synthesis and maturation. Stress to the ER triggers the unfolded protein response (UPR) and activates the three-branched pathways to restore cellular homeostasis. This includes the protein kinase RNA-like ER kinase (PERK) that plays a major role in the attenuation of global translation via phosphorylation of the eukaryotic translation initiation factor 2 alpha (eIF2α) at serine 51 [15]. However, the translation of a sub-set of mRNAs like *ATF4, CHOP* and *p53*, which encode for factors mediating the ER stress response are selectively stimulated. Short upstream open reading frames (uORFs) present in the 5’ of the *ATF4* and *CHOP* mRNAs have been attributed to the PERK-mediated alterative mode of translation initiation [16, 17], but the molecular mechanism remains elusive [5].

Structural dynamics of proteins and RNAs in response to signaling pathways are highly anticipated to play important roles in cell biology but are yet difficult to study. Here, we have addressed the molecular mechanism of PERK-mediated p47 induction and the evolution of the p53-dependent ER stress-response pathway. We show that PERK activity leads to changes in the human *p53* mRNA structure that are required and sufficient for cap-independent translation initiation of p47. These structural changes do not take place in the murine *p53* mRNA. Together with previous works on alternative *p53* mRNA structural changes imposed by the ATM kinase following DNA damage resulting in full-length p53 activation [18], these data emphasize the importance of regulated RNA structures in cellular pathways and how the formation of specific structures has co-evolved with the function of the encoded protein.

## Materials and methods

### Cell culture, transfection, and treatments

p53-null H1299 cells (non-small-cell lung carcinoma human cell line) was mostly used for experimental analysis unless mentioned otherwise. Other cell lines used were p53-null and p53-WT mouse embryonic fibroblasts (MEF) cells and the A549 (p53-WT) cell line. Cells were cultured in RPMI 1640 medium (31870074, Thermo Fisher Scientific) supplemented with 10% fetal bovine serum (A3160502, Thermo Fisher Scientific), 100 U ml^−1^ penicillin and 100 mg ml^−1^ streptomycin (15140122, Thermo Fisher Scientific) and 2 mM L-glutamine (25030081, Thermo Fisher Scientific) and maintained at 37 °C in a humidified 5% CO_2_ incubator. Cell lines were routinely checked for mycoplasma contamination using MycoStrip™—Mycoplasma Detection Kit (rep-mys-10, Invivogen). Plasmid DNA transfections were performed using GeneJuice reagent (70967, Sigma-Aldrich) following the manufacturer’s protocol. siRNAs targeting hnRNPC1/C2, eIF2α, and PTB, and AllStars negative control siRNA (Qiagen, Valencia, CA, USA) were transfected using HiPerFect reagent (301704, Qiagen) following manufacturer’s instructions. Efficiency of siRNAs was assessed by Western blot analysis. To induce ER stress, cells were treated with 100 nM concentration of thapsigargin (Thap) (T7459, Thermo Fisher Scientific), or tunicamycin (T7765, Sigma-Aldrich) prepared in Dimethyl sulfoxide (DMSO) (276855, Sigma-Aldrich) for 16 h, unless specified otherwise.

### Plasmid constructs

All constructs were generated using the pcDNA3 eukaryotic expression vector (Life Technologies, Carlsbad, CA, USA) unless stated otherwise. p53-WT and p53/47 constructs have been described previously [5, 19]. Mutations inserted into the human and mouse p53 sequences were carried out using site-directed mutagenesis or PCR-based cloning. Hybrid-p53 constructs were generated using the custom gene synthesis services from Proteogenix, France, and cloned into the pcDNA3 vector.

### Western blotting

Cells were washed with ice-cold phosphate buffer saline and lysed in RIPA buffer (Thermo Fisher Scientific), supplemented with a complete protease inhibitor cocktail (Roche, Basel, Switzerland). Equal protein amounts were resolved in 10% Bis–Tris Plus Gels (Thermo Fisher Scientific), transferred onto the BioTrace NT pure nitrocellulose blotting membrane (PALL Corporation) and blocked with 5% non-fat dry milk in Tris-buffered saline pH 7.6 containing 0.1% Tween-20. Proteins were probed with corresponding antibodies (listed below) and detection was performed using WestDura (Thermo Fisher Scientific) with myECL Imager (Thermo Fisher Scientific). Antibodies: anti-p53 rabbit pAbs (CM-1-Recamo); anti-actin mouse pAbs (AC-15, Sigma-Aldrich), anti-hnRNP C1/C2 (sc-32308, Santa Cruz), anti-PTB (32–4800, Thermo Fisher Scientific), anti-eIF2α (sc-133132, Santa Cruz) HRP-conjugated secondary antibodies (Dako, Glostrup, Denmark). Western blots represent *n* ≥ 3 and the original uncropped blots are provided in the Supplementary Information.

### RNA isolation and qRT-PCR

The total RNA was purified from MEF cells or H1299 cells post-transfection using the RNeasy Mini Kit (74104, Qiagen) following the manufacturer’s protocol. RT was carried out using Superscript II Reverse Transcriptase (18064014, Thermo Fisher Scientific) and oligo(dT) primers (18418012, Thermo Fisher Scientific). RT-qPCR was performed on QuantStudio™ real-time PCR system (Applied Biosystems) using PowerUp™ SYBR™ Green Master Mix (A25741, Thermo Fisher Scientific). Primer sequences: 14-3-3-σ, forward 5’-TGCTGGACAGCCACCTCATCAA; reverse 5’-GGCTGAGTCAATGATGCGCTTC. Actin, forward 5’-TCACCCACACTGTGCCCATCTACGA-3’; reverse 5’-TGAGGTAGTCAGTCAGGTCCCG-3’.

### Flow cytometry

Fluorescence-activated cell sorting (FACS) was performed as described in [20]. Briefly, murine MEFs (p53-WT) treated with DMSO/Thap were fixed by overnight incubation with cold 70% ethanol and treated with RNAse A (Sigma-aldrich), cells stained with propidium iodide were then analyzed with an LSR flow cytometer and CellQuest software (Becton-Dickinson).

### In-cell RNA SHAPE-MaP

The RNA SHAPE-MaP was performed with the protocol adapted from the methods published by the Weeks group [21, 22]. Briefly, H1299 cells grown in 6-well plates were transiently transfected with indicated constructs and treated with the indicated conditions (DMSO/Thap). 36 h post-transfection, cells were washed with PBS and added 900 µl of RPMI media. The SHAPE reagent 1-Methyl-7-nitroisatoic anhydride (1M7) (Sigma-Aldrich) was added to a final concentration of 10 mM by adding 100 µl of 100 mM 1M7 to 900 µl of RPMI media and treated for ~90 s at 37 °C. The same volume of DMSO was added to the untreated samples. Cells were then washed with PBS and harvested. RNA purification was carried out using the RNeasy kit (Qiagen), followed by DNAse I digestion for 30 min at 37 °C. Reverse-transcription of purified RNA was carried out with the primers indicated in Supplementary Table S1, using the MaP buffer and Superscript II Reverse Transcriptase (Thermo Fisher Scientific). Synthesized cDNAs were then purified and amplified using Q5 DNA polymerase (NEB) using the indicated p53 primers. The PCR product was purified and quantified with a Qubit fluorometer and diluted to 0.2 ng/µl. Purified amplicons were then tagmented and a library was created using the Ilumina Nextera PCR library kit. Products from library PCR were then purified with the Agencourt AMPure XP beads (Beckman Coulter Life Sciences) as described in [22]. Library concentration was measured with a Qubit fluorometer and the size distribution was measured using an Agilent 2100 Bioanalyzer according to the manufacturer’s instructions. Libraries were then sequenced with the Paired-end 150 NovaSeq System (NovaSeq PE150, Novogene, UK). SHAPE reactivity profiles and comparisons were generated using the ShapeMapper 2 and deltaSHAPE scripts, using default settings as described in [22] and aligned to indicated p53 coding sequences (CDS). SHAPE-Map data shown are representative of at least two independent biological repeats and the deviations across biological repeats of each experiments were examined by the Spearman’s rank-order correlation coefficient.

### In vivo DMS footprinting

H1299 cells grown in 100 mm dishes were transiently transfected with the indicated constructs. In vivo dimethyl sulfate (DMS)-based modification of RNA was carried out as described previously [23]. Briefly, 36 h post-transfection, cells were washed and resuspended with PBS (100 µl). In total, 1 µl of DMS was added to the cell suspension and incubated for 2 min at room temperature with gentle shaking. After stopping the reaction with β-mercaptoethanol (10 µl), total RNA was extracted using TRIzol (Invitrogen). As a control, transfected cells were processed in the same way but without DMS. For primer extension, total modified RNA was denatured for 3 min at 95 °C and reverse transcribed using 3 units of AMV RT (Promega) along with 4 mM dNTPs and a ^32^P end-labeled primer annealing to the coding region of the *p53* mRNA for 1 h at 42 °C. The cDNA products were then analyzed on denaturing 8% acrylamide and 8 M urea gels along with a reference sequencing reaction generated with the ThermoSequenase® cycle sequencing kit (Amersham Biosciences) using the same end-labeled primer, followed by phosphorimaging analysis.

### Conservation analysis

*mRNA s*equences of mouse (*Mus musculus*), human (*Homo sapiens*), rhesus monkey (*Macaca mulatta*), cow (*Bos taurus*), dog (Canis *familiaris*), and pig (*Sus scrofa*) p53 were obtained from GenBank® (http://www.ncbi.nlm.nih.gov) and alignment was performed using Clustal Omega [24]. Consensus RNA secondary structure of the aligned p53 sequences were generated using RNAalifold server in ViennaRNA web suite [25].

### Statistical analysis

Statistical significance was analyzed by comparing data sets with corresponding reference points using two-tailed unpaired *t*-test (*p < 0.05; **p < 0.01; ***p < 0.001; ns: not significant). Spearman’s rank-order correlation coefficient was used to assess the biological replicates of RNA SHAPE-MaP data sets. Statistical assessments were performed using the GraphPad Prism software and Python.

## Results

### The mouse *p53* mRNA does not express the alternatively initiated p53 isoform

**Fig. 1:**
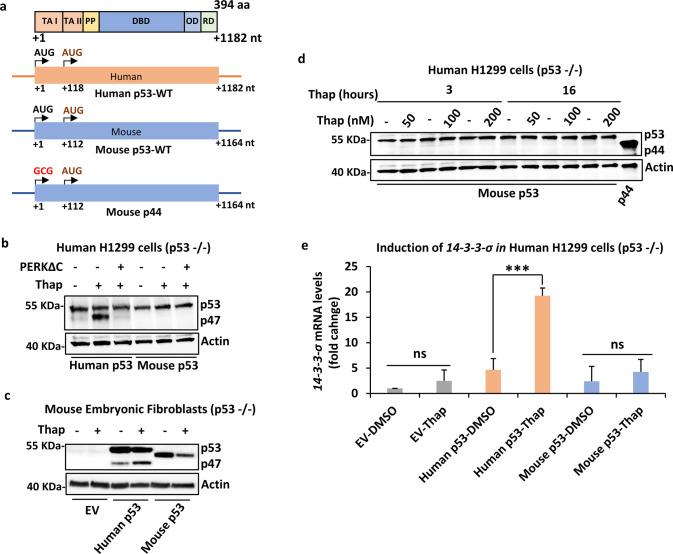
Mouse p53 does not express the p44 isoform during ER stress. **a** Cartoon depicting the functional domains of p53 protein, beginning with the two N-terminal transactivation domains (TAs) followed by the polyproline domain (PP), the DNA binding domain (DBD), the oligomerization domain (OD) and the C-terminal regulatory domain (RD). Below is the schematics of human (human p53-WT) and mouse (mouse p53-WT) p53 CDS and the corresponding mouse p53 message with a substitution of the first AUG > GCG (Mouse p44). The second in-frame AUG at +118 and +112, respectively, are indicated. **b** The Western blot shows the expression of human and mouse p53 isoforms in p53-null human H1299 cells under normal conditions and with ER stress induced by thapsigargin (Thap) treatment. The PERK construct lacking the C-terminus (PERKΔC) is a dominant-negative mutant and prevents ER stress-induced p47 expression. **c** Expression of human and mouse p53 constructs in p53-null mouse embryonic fibroblasts, under normal and ER stress conditions. **d** Increasing the time and dose of Thap does not result in the p44 isoform expression. The mouse p44 was not induced with Thap treatment and only following mutation in the first AUG (AUG > GCG). **e** The graph showing the expression levels of *14-3-3-σ* mRNA under the indicated conditions. EV-empty vector control. Actin was used as a loading control. For RT-qPCR graph, the mean of three independent experiments were shown with s.d. Statistical significance was calculated using unpaired *t-*test (***p < 0.001; **p < 0.05; *p < 0.1; ns not significant).

We and others have shown that the human p53 isoform p47 is induced by cap-independent mRNA translation at the second in-frame AUG at position +118 during stress to the endoplasmic reticulum (Fig. 1a) [7, 8]. The PERK-dependent induction of p47 from the human *p53* message was abrogated by over-expressing the dominant-negative PERK construct that lacks the C-terminal domain (PERKΔC), as expected [5]. We tested if the mouse p53 isoform p44 could also be regulated in a similar fashion. Surprisingly, despite having an AUG in the same position, we observed no expression of p44 from the murine p53 cDNA either in the presence, or absence, of thapsigargin (Thap)-induced ER stress in human H1299 (p53-null) cells (Fig. 1b). Similar results were obtained using the ER stress-inducing reagent tunicamycin (Fig. S1a) or when the human or mouse p53 constructs were expressed in p53 null mouse embryonic fibroblasts (Fig. 1c). Hence, p47 expression from the human p53 cDNA is promoted by ER stress independently of which species the cells originate from. To test if the induction of p44 required different levels of ER stress, we used Thap concentrations of 50 to 200 nM for either 3 or 16 h. But neither the dose nor the time resulted in the p44 expression. Replacing the +1 AUG with GCG in the mouse p53 prevented full-length p53 expression and resulted in the expression of the p44 isoform from the second in-frame AUG, confirming that the antibodies used recognized p44 (Fig. 1d). Next, we assessed the cell cycle status under normal and ER stress conditions using the FACS analysis. We have previously shown that human p53 upon ER stress stimulates the *14-3-3-σ* via p47 expression and causes G2-cell cycle arrest [5]. However, in human H1299 cells transfected with mouse p53 we did not observe any significant induction of *14-3-3-σ* mRNA following ER stress (Fig. 1e). In line with this, murine MEFs (p53-WT) did not show any increase in G2 arrest following ER stress (Fig. S1b). These results show that the murine *p53* mRNA does not allow PERK-mediated induction of initiation at the second AUG and that the p53-dependent ER stress-response pathway is not present in mice.

### ER stress causes RNA structural changes in human p53 both upstream and downstream of second AUG

**Fig. 2:**
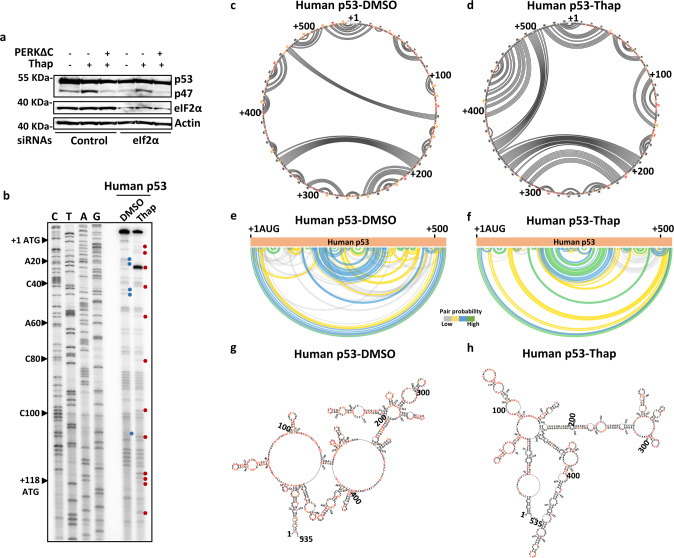
ER stress induces significant structural alterations in human *p53* mRNA. **a** Western blot showing the expression of p53 and p47 isoforms under indicated conditions. Silencing of eIF2α has no effect on PERK-mediated p47 translation during ER stress (see also Fig. S2). **b** DMS footprint shows the methylation pattern of unpaired adenine or cytosine residues of human *p53* mRNA under normal (DMSO) and ER stress (Thap) conditions. The differences in DMS-footprint patterns indicate changes in the *p53* mRNA structure following ER stress. Unpaired regions in the ER stress conditions are indicated in red dots and in normal conditions in blue. Circular plots showing the base-pairing potentials of the human *p53* mRNA CDS based on the SHAPE reactivity under normal (DMSO) (**c**) and ER stress (Thap) conditions (**d**). Base-pairing across the nucleotides are indicated by lines, and modifications in the lining pattern indicate the RNA structural alterations. Arc plots showing the probability of base pairings observed in the circular plots (**c** and **d**) under normal (**e**) and ER stress conditions (**f**), highly probable base-pairings are indicated in green color. Secondary structure of the *p53* mRNA coding sequences from +1 to +535 nt mapped using the SuperFold algorithm based on the SHAPE values under normal (**g**) and ER stress conditions (**h**), SHAPE modified nucleotide sequences are indicated in orange and red.

We next sought to understand how PERK kinase regulates species-specific mRNA translation initiation during ER stress and we first examined whether the induction of p47 was affected by PERK-dependent eIF2α phosphorylation. However, neither silencing of eIF2α, nor over-expression of phosphorylation mutant eIF2α (S51A), had any significant effects on the induction of p47 during ER stress. Furthermore, the status of eIF2α phosphorylation did not affect the dominant-negative effect of the PERKΔC on p47 synthesis, suggesting that the regulation of the alternative translation initiation of the *p53* mRNA by PERK is independent of eIF2α phosphorylation (Figs. 2a and S2). Next, we tested if alternative initiation of translation involves structural regulation of the *p53* mRNA and we performed DMS-based RNA footprinting, using the reverse transcriptase (RT)-based p53 primer extension on RNAs extracted from the DMS-pulsed cells. DMS modifies the unpaired adenine and cytosine residues, and these modifications are visualized using RT pauses in the primer extension. When we compared the DMS footprint of the *p53* mRNA under normal (DMSO) or ER stress (Thap) conditions, we observed significant changes in the DMS footprint pattern in regions between +1 to +118 nts. Regions affected by ER stress are indicated with blue and red dots (Figs. 2b and S3). Since DMS-based footprinting does not cover structures of large RNA regions and has nucleotide bias, we next employed the high-throughput in-cell RNA structural probing using the selective 2′-hydroxyl acylation analyzed by primer extension and mutational profiling (SHAPE-MaP) approach for high-accuracy comparative structural analysis of large RNA regions at single nucleotide resolution [21]. Following ER stress, we observed significant alterations in the base-pairing potentials in the 5’ part of the human *p53* CDS, as indicated in the circular plots (Fig. 2c, d) and in the arc plots (Fig. 2e, f). Secondary structures predicted by the SuperFold algorithm based on SHAPE reactivity illustrate these changes (Figs. 2g, h and S4). The SHAPE data support the DMS-footprinting and show that the 5’ part of the *p53* CDS RNA becomes structurally altered during the ER stress. Interestingly, we also observed SHAPE reactivity variations in the regions of +210 -> +403 nts under the ER stress conditions, and accordingly, SuperFold indicated significant structural alterations downstream of the 2nd AUG (+118). Altogether, these results indicate that ER stress induces structural alterations of the *p53* mRNA, that involve regions both upstream and downstream of the 2nd AUG.

### Mouse *p53* RNA does not undergo structural alterations during ER stress

**Fig. 3:**
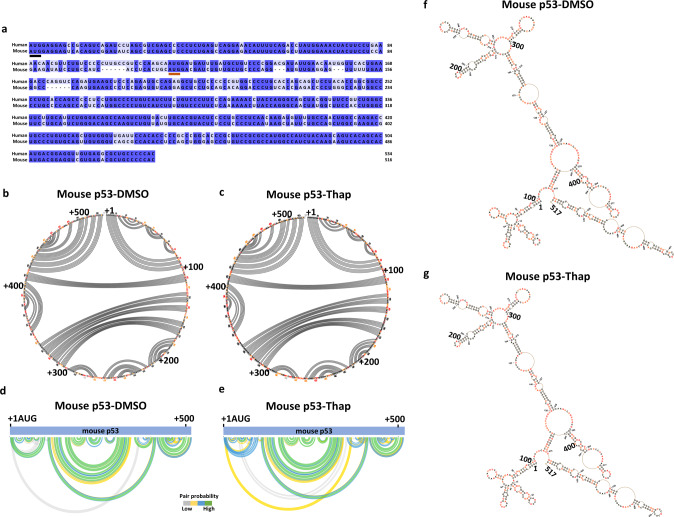
Mouse *p53* mRNA structure was not altered during ER stress. **a** Nucleotide sequence alignment of human (+1 to +535 nts) and mouse (+1 to +517 nts) *p53* mRNA CDS, positions of 1st and 2nd AUGs are marked with lines. Circular plots of the mouse *p53* mRNA base-pairing potentials based on the SHAPE reactivity under normal (DMSO) (**b**) and ER stress (Thap) conditions (**c**). Arc plots show the probability of base pairings observed in circular plots (**a** and **b**) under normal (**d**) and ER stress conditions (**e**). Secondary structures of the mouse *p53* mRNA coding sequences from +1 to +517 nts mapped using the SuperFold algorithm based on the SHAPE values under normal (**f**) and ER stress conditions (**g**), SHAPE modified nucleotides are indicated in orange and red.

Since mouse p53 cDNA does not induce the p44 isoform, we sought to determine if mouse *p53* RNA structures are affected by ER stress by performing the RNA SHAPE-MaP. Nucleotide sequence alignments between human, mouse, and across different species, shows that the first 80 nts (+1 to +80) of the p53 CDS are highly conserved, followed by variable bases until +248 nts and conserved sequences further downstream (Figs. 3a and S5). In agreement to this, alignment-based consensus secondary structure prediction using the RNAalifold web server [25, 26] showed the conservation of RNA secondary structure in the first 80 nucleotides of p53 CDS, across the spectrum of different species analyzed (Fig. S6). We analyzed the mouse *p53* RNA structure from cells under normal and ER stress conditions. Unlike human *p53*, mouse *p53* mRNA did only show minor changes in the base-pairing potential under the ER stress conditions, as indicated in the circular plots (Fig. 3b, c) and in the arc plots (Fig. 3d, e). In line with this, SuperFold based on SHAPE reactivity profiles did not alter much between the normal and ER stress conditions (Figs. 3f, g and S7). Hence, the mouse *p53* mRNA structure is not affected by ER stress, which can help to explain why murine *p53* does not express p44.

### PERK mediates ER stress-induced structural alterations in the human *p53* mRNA

**Fig. 4:**
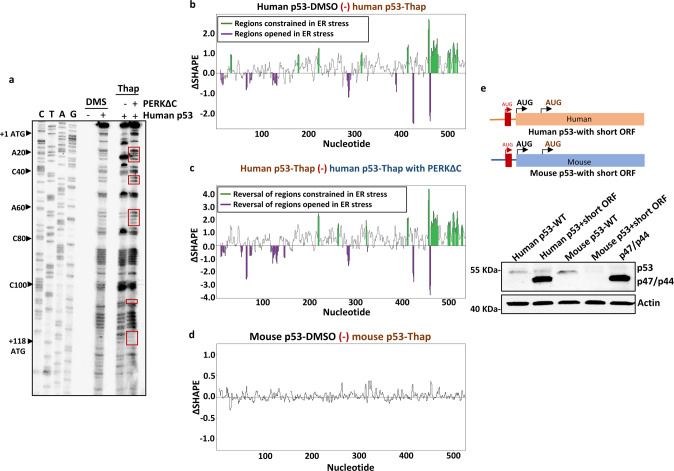
PERK regulates the structural changes of human *p53* mRNA under ER stress. **a** DMS-footprint of human *p53* mRNA under normal, ER stress and ER stress with PERKΔC over-expression. The presence of the dominant-negative PERKΔC reverses the methylation pattern, indicating that the changes in the *p53* mRNA structure following ER stress are PERK-dependent. Modifications reversed by PERKΔC are marked with red boxes. **b** ΔSHAPE analysis demonstrating significant structural alterations in *p53* mRNA following the ER stress (Thap), with RNA regions constrained during the stress are highlighted in green and the regions opened or exposed in violet. **c** ΔSHAPE analysis demonstrating that over-expression of dominant-negative mutant PERKΔC reverses the structural alterations generated during ER stress. **d** ΔSHAPE analysis of mouse *p53* mRNA did not show structural alterations following the ER stress. **e** Top panel: cartoon depicting human and mouse p53-WT constructs with a short ORF inserted upstream of the 1st AUG. Bottom panel: Western blot showing the expression of p53 and p47/p44 isoforms with the indicated constructs.

We next set out to test if the structural changes in the human *p53* mRNA following ER stress are PERK-dependent. For this reason, we over-expressed a dominant-negative PERKΔC in cells undergoing ER stress and compared the structural alterations. Results using DMS-based footprinting showed that the Thap-induced structural changes between the 1st and 2nd AUGs were reversed in the presence of PERKΔC (indicated in red boxes) (Fig. 4a). Furthermore, we also performed SHAPE-MaP and analyzed the differences in the structural alterations using the delta SHAPE (ΔSHAPE) script. The ΔSHAPE analysis allows us to compare the differences in SHAPE reactivity between any two states/conditions. We used ΔSHAPE to compare the structural differences of human *p53* mRNA between the normal (DMSO) and ER stress (Thap) conditions (Fig. 4b), with or without the presence of PERKΔC (Fig. 4c). Results showed that most, if not all, SHAPE reactivities generated under ER stress conditions were reversed in the presence of PERKΔC (Fig. S8). Hence, ER stress-induced changes in *p53* mRNA structures can be attributed to the functional activity of PERK. ΔSHAPE analysis of the mouse *p53* mRNA under normal (DMSO) and ER stress (Thap) conditions did not show any significant differences (Fig. 4d). Importantly, the results from the DMS-based RNA footprinting are mostly in agreement with the ΔSHAPE analysis. The reproducibility of SHAPE experiment was assessed by Spearman’s correlation analysis, using the data from independent biological replicates of all the RNA-SHAPE-MaP experiments. Results indicated the excellent agreement across independent replicates with Spearman *R* > 0.9 for all data sets of SHAPE experiment (Fig. S9).

Introducing a short ORF upfront of the 1st AUG in both human and mouse *p53* reading frames abrogated translation initiation from the 1st AUG in both mRNAs under normal conditions. However, only in the human message was this accompanied by an initiation from the 2nd AUG, further illustrating the differences between the human and murine *p53* mRNAs (Fig. 4e). Trans-acting factors that bind the *p53* mRNA have been reported, including heterogeneous ribonucleoprotein (hnRNP) C1/2 and the polypyrimidine tract binding protein (PTB) [27, 28]. Knockdown of hnRNPC1/2 resulted in reduction of p47 expression under normal and ER stress conditions but failed to abrogate the ER stress-mediated induction of p47. PTB has been shown to promote initiation from the 2nd AUG in a bi-cistronic luciferase reporter construct [28] but, likewise, the suppression of PTB had little effect on ER stress-mediated induction of p47 (Fig. S10).

Together with the observation that eIF2α does not play a role in p47 expression, these results show that PERK-mediated structural alterations of the human *p53* mRNA are essential and sufficient for cap-independent translation initiation from the second AUG at +118.

### ER stress-response elements controlling p47 expression include sequences downstream of the p47 initiation codon

**Fig. 5:**
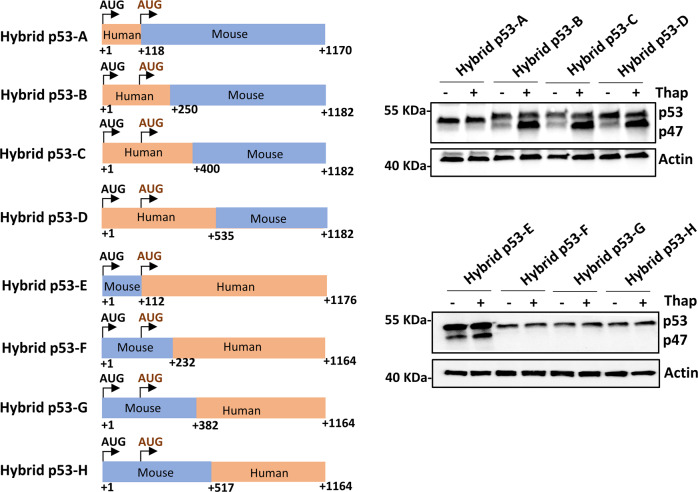
ER stress response element requires RNA sequences downstream of the second AUG in human p53. Left panel: cartoons depicting the design of the human-mouse hybrid p53 constructs. Right panel: Western blots showing the expression of these constructs in H1299 cells. Initiation from the 2nd AUG requires human *p53* mRNA sequences extending beyond the 2nd AUG. Sequences including downstream of the 2nd AUG in the human *p53* mRNA (+118 to +250 nts) are required to induce initiation at the second AUG.

It was previously shown that deletion of the +1 to +118 nts of the p53 coding sequence prevents ER stress-mediated expression of p47 and, thus, this sequence was attributed to containing the information required to control p53 isoform expression during ER stress conditions. However, SHAPE data (see Fig. 2) showed that ER stress resulted in altered structures both upstream and downstream of the 2nd AUG of the human *p53* message. We wanted to know if sequences downstream of +118 play a role in mediating p47 induction. For this reason, we swapped the sequences between the human and mouse p53 and generated hybrid p53 constructs and expressed these under normal or ER stress conditions. Surprisingly, fusing the +1 to +118 of human p53 to the mouse p53 sequence starting at the 2nd AUG (+112 to +1170) (hybrid p53-A) did not result in the induction of p44. Switching the mouse +1 to +112 in front of the human *p53* (+118 to +1176) (hybrid p53-E) still allowed the induction of the p47 isoform following ER stress (Fig. 5). The first 80 nucleotides are rather conserved between mouse and human, with only eight mismatch codons with 12 nucleotides and 5 amino acid differences. Consequent exchange of nucleotides in the human sequence in codons 4, 6, 8, 10 12, 15, 17, 21, and 22 with the corresponding mouse codons had little, or no effect, on the induction of p47 following ER stress. Furthermore, deletion of the variable region in human (+83 to +118) and mouse (+83 to +112) p53 coding sequences upstream of the 2nd AUG had no effect on isoform synthesis after ER stress, implying that the regions downstream of the 2nd AUG are required for p47 induction (Fig. S11). We created additional p53 hybrid constructs by swapping +1 to +250 nts (hybrids p53-B and F), +1 to +400 nts (hybrids p53-C and G), and +1 to +535 nts (hybrids p53-D and H) between human and mouse p53, in order to find the minimal region of the ER stress response element in the *p53* mRNA essential for p47 induction (Fig. 5). The exchange of +1 to +250 nts (hybrid p53-B) from human to mouse p53 showed p47 induction under ER stress conditions, whereas the corresponding exchange of nucleotides from mouse to human p53 (hybrid p53-F) loses the ability to induce p47. Likewise, we observed the p47 induction with hybrid p53-C (+1 to +400 nts) and hybrid p53-D (+1 to +535 nts) constructs under ER stress conditions and no p47 induction with hybrid p53-G (+1 to +382 nts) and hybrid p53-H (+1 to +517 nts) constructs bearing the corresponding exchange of mouse sequences to human p53. Together with the SHAPE data, these results show that the ER stress-response elements are located downstream of the 2nd AUG in the region +118 to +250 nts of human *p53* mRNA.

## Discussion

The p47 was the first human p53 isoform identified and is derived from an alternative mode of cap-independent mRNA translation initiation from the 2nd in-frame AUG at codon 40 following ER stress [5]. The equivalent isoform in mice, p44, was detected following retrovirus infection [9]. It was expected that p44 would also be expressed from alternative mRNA translation initiation since it carries an AUG codon in a similar position as the human message, but the murine *p53* mRNA does not give rise to p44 under ER stress conditions, neither in mouse nor in human cells. This can be explained by the observation that murine *p53* mRNA does not undergo the structural changes required to initiate alternative translation in response to PERK activation. An insertion of a short ORF upstream of the human and murine *p53* mRNAs results in the suppression of both full-length proteins, but only the human *p53* mRNA responded with an induction of p47, demonstrating that the structural changes in the *p53* mRNA are required and sufficient for p47 induction. Furthermore, ER stress-mediated structural changes are reversed by introducing a dominant-negative PERK, showing that PERK activity alone mediates ER stress-dependent changes in the RNA structure.

It is widely anticipated that structural changes in proteins and RNAs play a key role in the response to signaling pathways. Cryo-EM studies allow us to envision larger structures, but these are in vitro analysis and do not reflect the dynamics of structures or interactions in cell. SHAPE, on the other hand, allows comparison of structural differences in mRNAs under different cellular conditions. For example, we observed that PERK affects structures downstream of the 2nd AUG, and subsequently, we could show that indeed, these structures are required for PERK-mediated p47 induction. It is interesting that a large proportion of the *p53* mRNA is affected by PERK and this illustrates how separate and long-distance regions affect the dynamics of mRNA structures. Another example comes from the binding of nucleolin to G-quadruplex (G4) structures in the coding sequence of the *EBNA1* mRNA. Changing the 5’ UTR of the *EBNA1* message, or moving the G4s throughout the coding sequence, disturbs the G4 structure and prevents nucleolin binding [29].

**Fig. 6:**
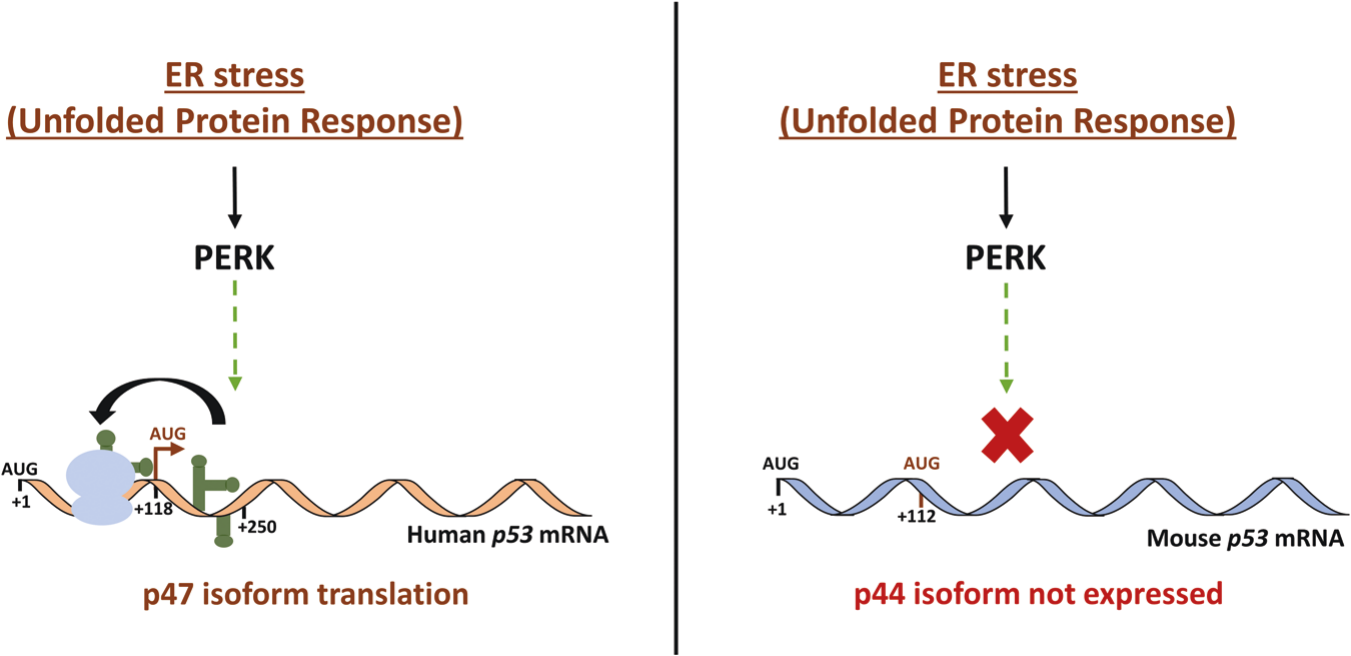
PERK-dependent RNA structural alterations control the translation of the human p47 isoform during ER stress. Stress to the endoplasmic reticulum (ER) activates the unfolded protein response (UPR) and PERK. PERK modulates the human *p53* mRNA structure to facilitate alternative translation initiation from the 2nd AUG and the expression of the p47 isoform, independently of eIF2α. PERK-induced RNA structures downstream of the 2nd AUG promote the formation of a ribosome entry site upstream of the 2nd AUG. The murine *p53* mRNA does not respond to PERK-mediated structural changes and the p44 isoform is not expressed. It is unknown which RNA chaperone(s) are responsible for PERK-induced RNA structural changes.

We do not yet know the cellular factor responsible for PERK’s effect on RNA folding. PERK-mediated translation initiation control of ER stress-response factors, such as ATF4, has been attributed to the phosphorylation of eIF2α and the suppression of the initiation of a short uORF of *ATF4* and the subsequent initiation of the main ORF [17]. However, the synthesis of p47 is independent of eIF2α phosphorylation. The differences in the two modes of PERK action might be related to the fact that p47 synthesis is cap-independent and reliant on changes in RNA structures downstream of the 2nd AUG that create a ribosome entry site upstream of the AUG (Fig. 6). Nevertheless, it will be interesting to see what other mRNAs are subject to PERK-mediated regulation of RNA structures.

PERK-mediated changes in *p53* mRNA structures can be compared with ATM kinase-mediated structural changes of the *p53* mRNA during the DNA damage response. But in the latter case, changes in RNA structures allow MDM2 to bind the RNA with the consequent induction of initiation at the 1st AUG and the activation of full-length p53 [30]. The *p53* mRNA structure required to interact with MDM2 evolved from being temperature regulated in the pre-vertebrate *Ciona intestinales* to become regulated by the DNA stress-response pathway in mammalian cells [31–33]. Thus, the dynamics of *p53* mRNA structures in response to different stresses have evolved independently and have variable effects on the function of the encoded p53 protein. The RNA structural changes required to activate p53 following DNA damage are prevented by cancer-derived single synonymous mutations [33–35] but as of today we do not know of synonymous mutations that affect the ER stress response pathway.

An interesting question is why human cells evolved the p53 ER stress-response. A p44 transgenic mouse on a p53 wild type background showed a progeroid phenotype with altered pluripotency of stem cells that depends on the presence of the full-length p53 [11]. This led to the notion that it is the relative ratio of the two isoforms that determines the p53 response. It was also shown that p47 is associated with human glioblastoma and with regenerative processes in neural tissue [13]. Studies have also linked p47 to neurodegenerative diseases such as Alzheimer’s by regulating the phosphorylation of tau [11]. The effect of p47 under ER stress conditions in vitro has been studied in some detail, and a p47-dependent G2 cell cycle arrest via the suppression of p21^CDKN1^ and the activation of 14-3-3σ facilitate ER repair [19]. If the ER stress is prolonged, p47 can promote apoptosis by suppressing synthesis of the ER chaperone BiP and the consequent activation of BIK [36]. It has been suggested that the lack of the N-terminal transactivation domain together with its RNA-binding capacity, renders p47 a transacting translation factor [12, 37, 38]. Neural cells are prone to gene expression control at the level of translation and it can be speculated that p47 plays a particular role in neural gene regulation [13, 39]. The p53 isoform (Δ133p53) lacks the first 132 amino acids and is abundantly expressed in early passages of normal human fibroblasts and at decreased levels in late passages and senescent cells [4]. The underlying molecular mechanism for this regulation is not known, but it illustrates that the expression of p53 isoforms with unique functional activities are not only species-specific but also controlled in a tissue-specific manner.

Taken together, this study shows that PERK promotes a species-specific *p53* mRNA folding that stimulates an alternative mode of translation initiation during the ER stress response that is independent of eIF2α. Further studies will show the frequency with which PERK regulates translation via alterations in mRNA structures.

## Supplementary information

Supplementary figures and table

Supplemental material_original Western blots

CDD_Checklist

## Data Availability

The authors declare that data supporting the findings of this study are available within the article and the Supplementary Information, or available from the corresponding authors upon reasonable request.
