# Supplementary material for: The p53 endoplasmic reticulum stress-response pathway evolved in humans but not in mice via PERK-regulated p53 mRNA structures
Source: Cell Death Differ. 2023 Feb 22;30(4):1072–81. doi: 10.1038/s41418-023-01127-y (PMC10070458; doi:10.1038/s41418-023-01127-y)
Supplement: Supplementary file 1 — Supplementary figures and table [file 41418_2023_1127_MOESM1_ESM.docx]

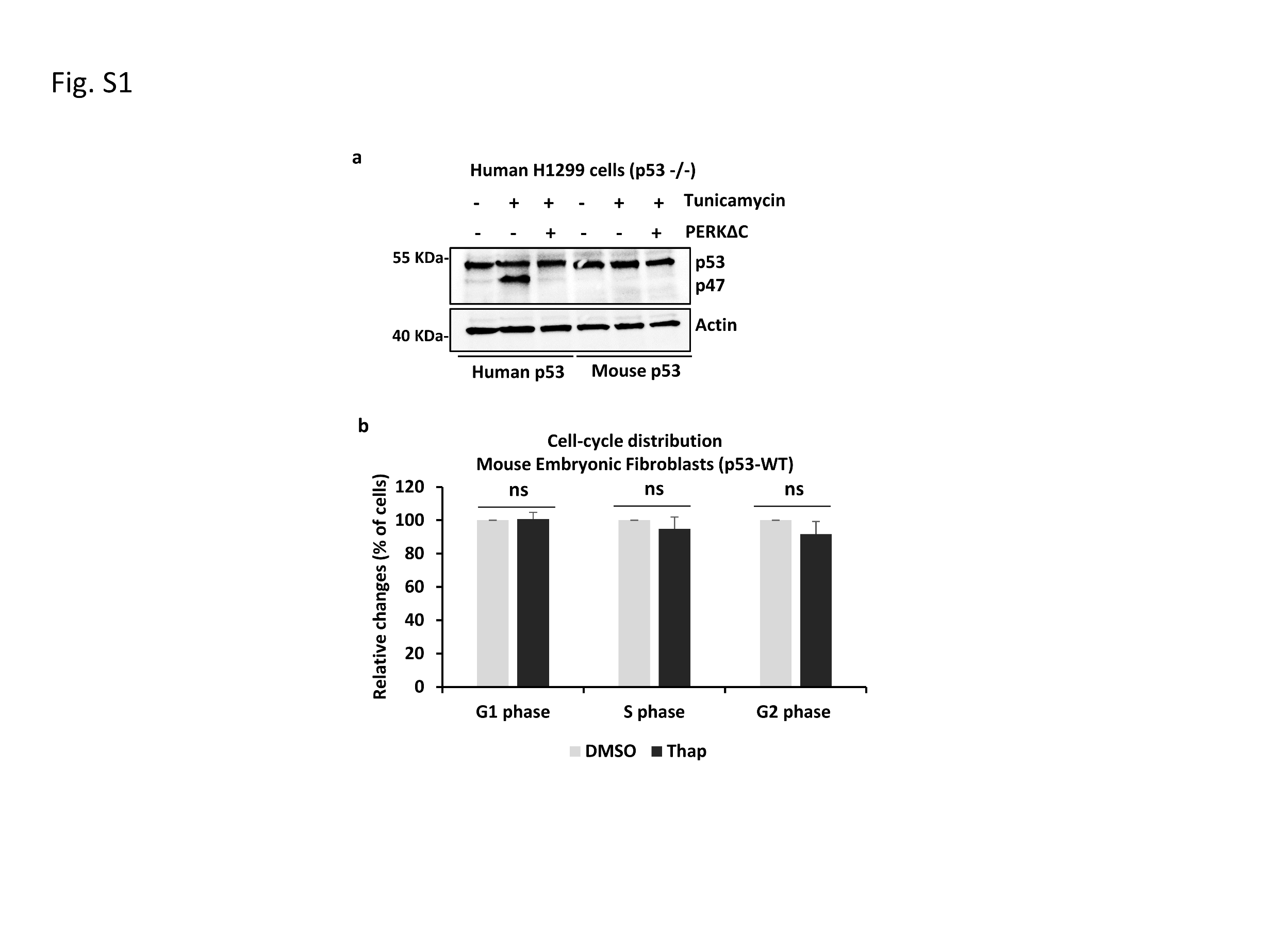


**Fig. S1. a)** Western blot shows the expression of human and mouse p53 isoforms in p53-null human H1299 cells under normal conditions and with ER stress induced by tunicamycin treatment. Actin was used as a loading control. **b)** Graph showing the distribution of cell-cycle phases in murine MEFs (p53-WT) under normal conditions and ER stress induced with Thap. For flow cytometry data, the mean of three independent experiments were shown with s.d. Statistical significance was calculated using t-tests (***p < 0.001; **p < 0.05; *p < 0.1; ns: not significant). Related to the main figure 1.


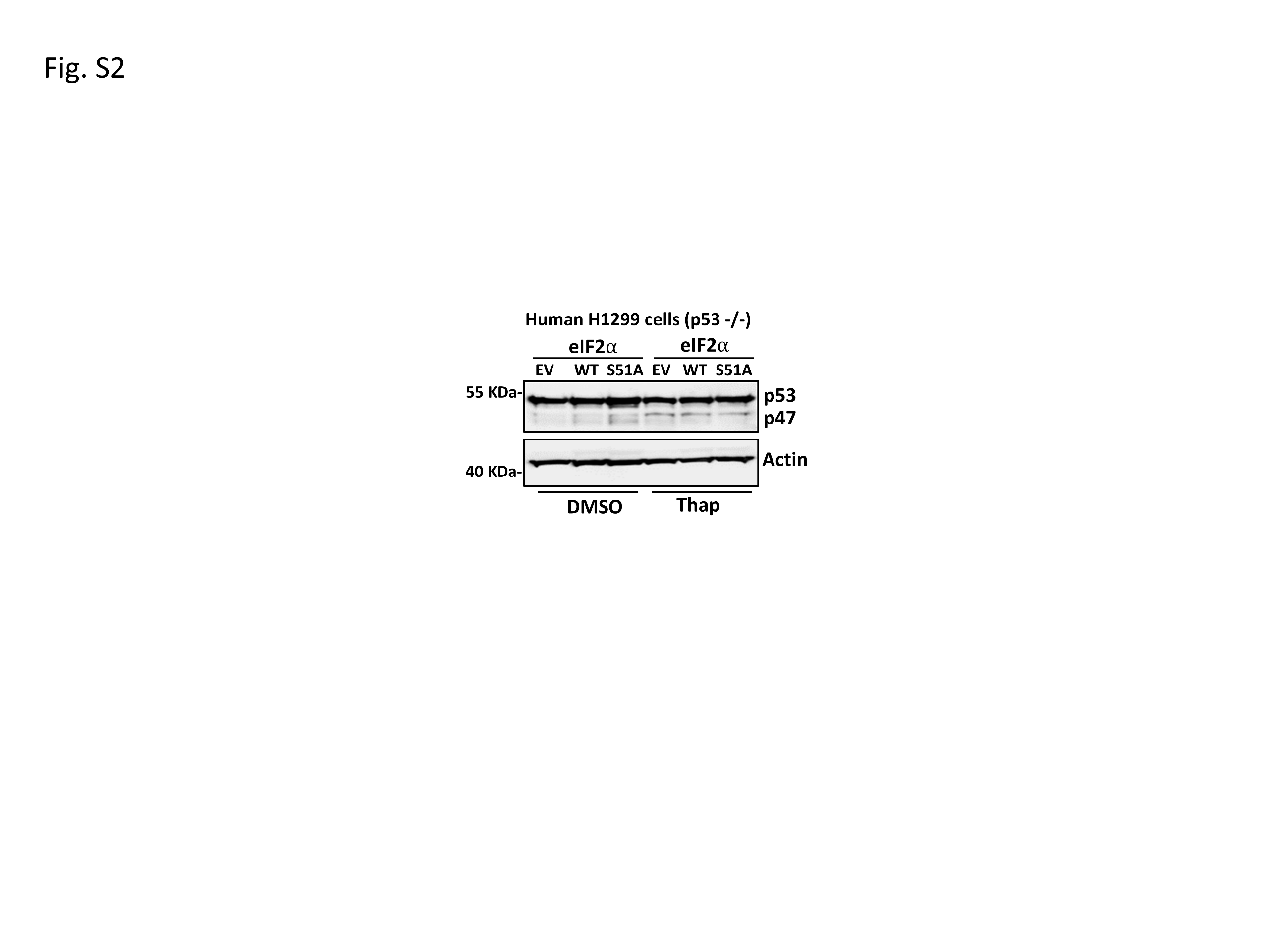


**Fig. S2.** eIF2α phosphorylation had no significant effect on the expression of p47. Western blot shows the expression levels of p53 and p47, with the over-expression of eIF2α-WT and or phosphorylation mutant eIF2α-S51A. p47 levels were not altered significantly either with the over-expression of eIF2α-WT or eIF2α-S51A. EV-vector control, actin was used as a loading control. Related to the main figure 2.


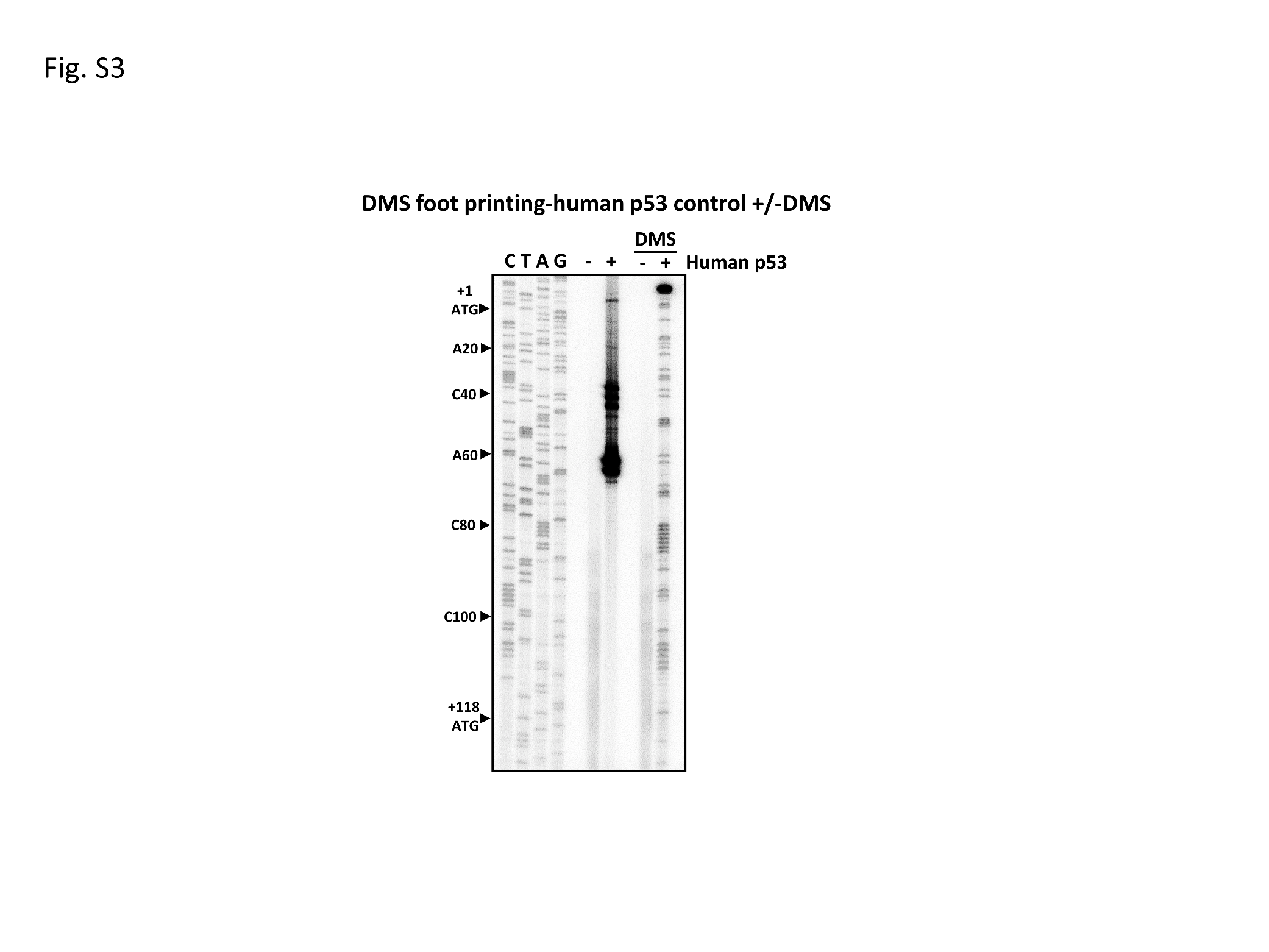


**Fig. S3.** DMS-based footprinting of 5’ coding sequences of the human *p53* mRNA. Autoradiograph shows RT pauses in the primer extension of the *p53* mRNA treated with or without DMS. H1299 cells expressing human p53 were pulsed, or not, for 2 min with 0.05% DMS. DMS modifies unpaired adenine and cytosine nucleotides, thereby causing a pause in primer extension. Related to the main figure 2.


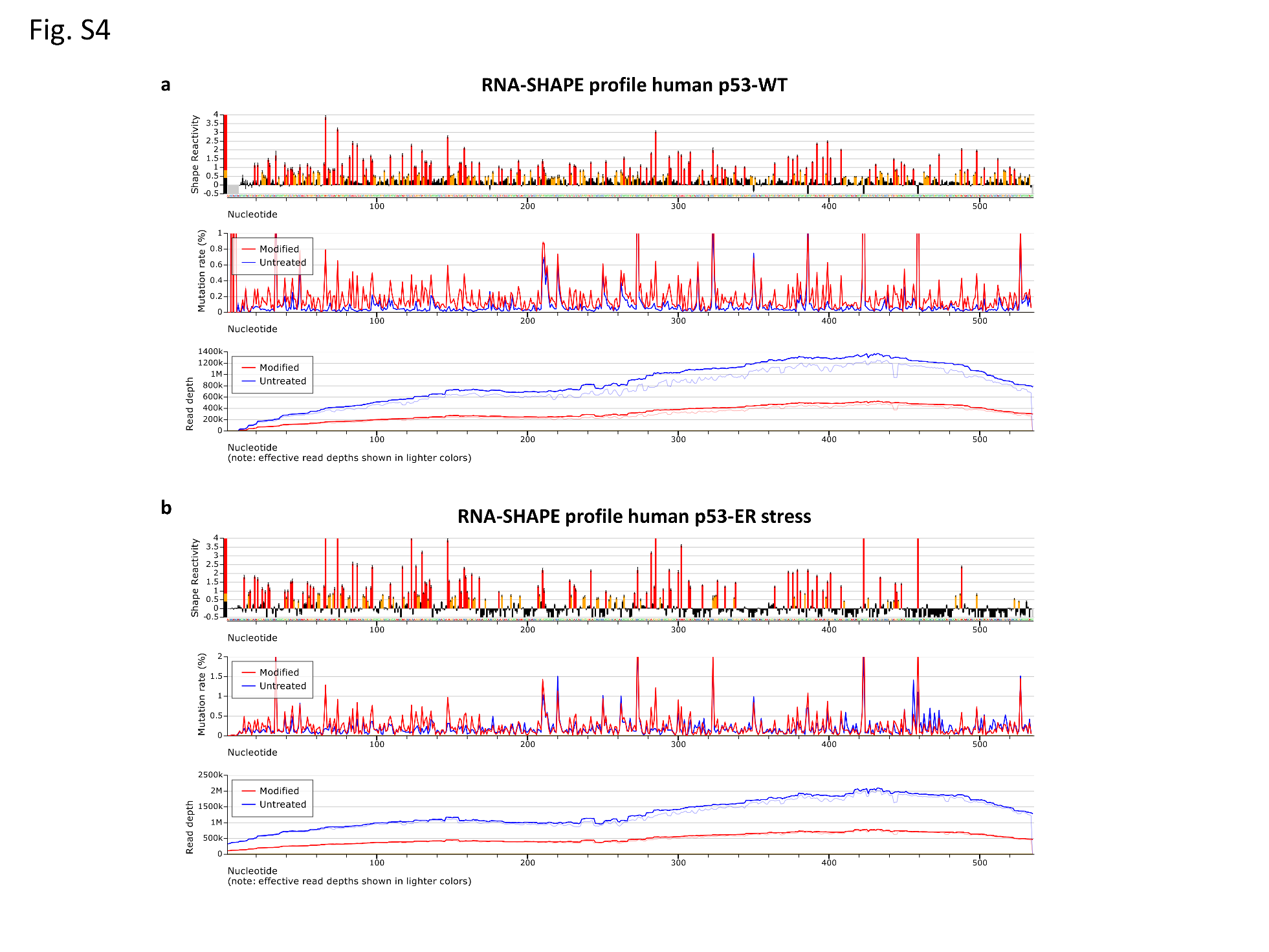


**Fig. S4.** RNA-SHAPE-MaP profiles of human *p53* mRNA under normal **(a)** and ER stress conditions **(b)**. The upper panel shows the SHAPE reactivity profiles, higher reactivities are indicated by red bars; the middle panel shows the mutation rate; and the lower panel shows the sequence read depth**.** RNA modified with 1M7 (modified) is indicated in red, and RNA treated with DMSO control (untreated) is indicated in blue. Related to the main figure 2.

**
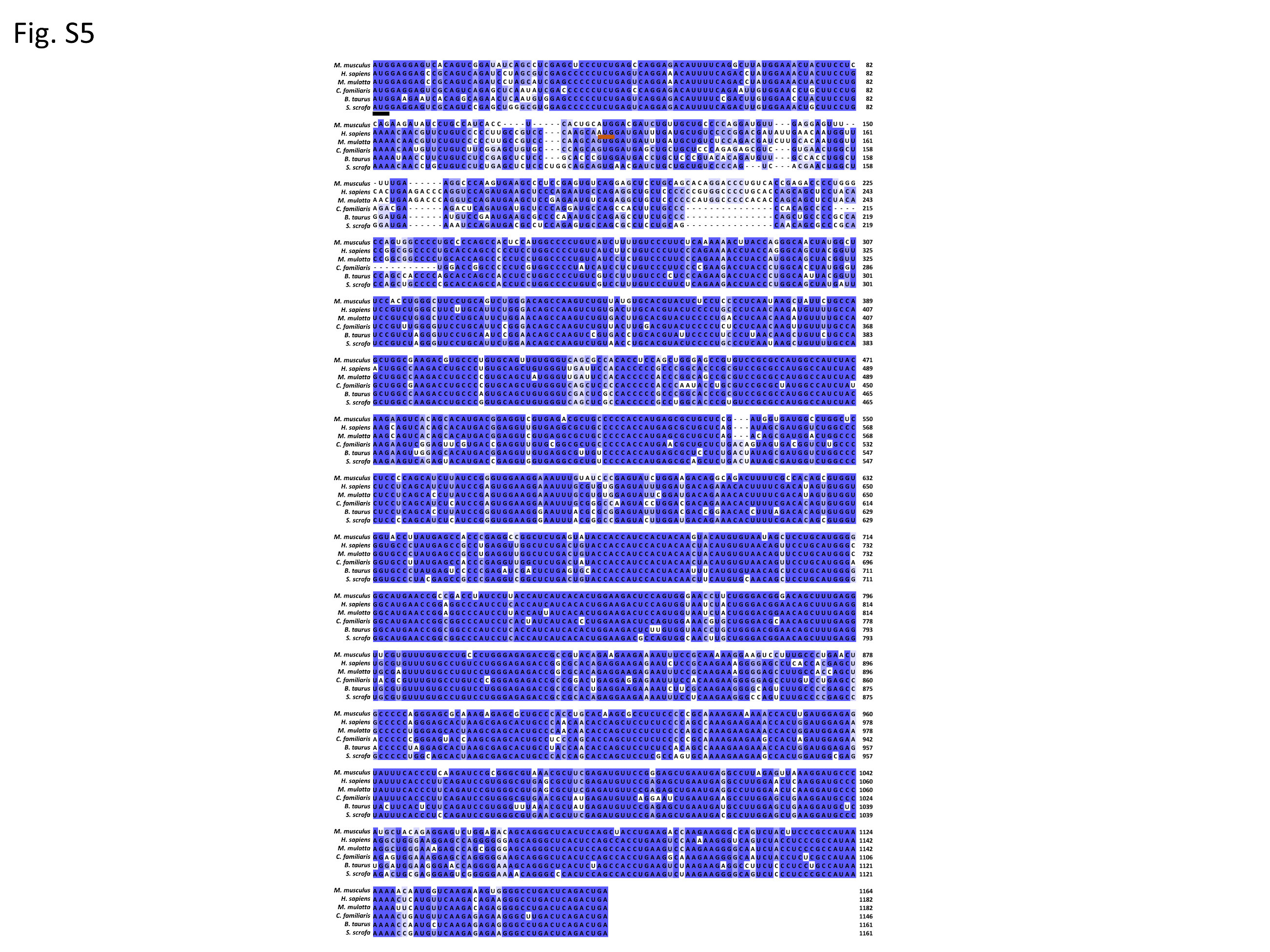
**

**Fig. S5.** Sequence alignment of *p53* mRNA CDS across multiple species, positions of the 1^st^ and 2^nd^ AUG are indicated with lines. Alignment was generated using Clustal Omega. Related to main figure 3a.


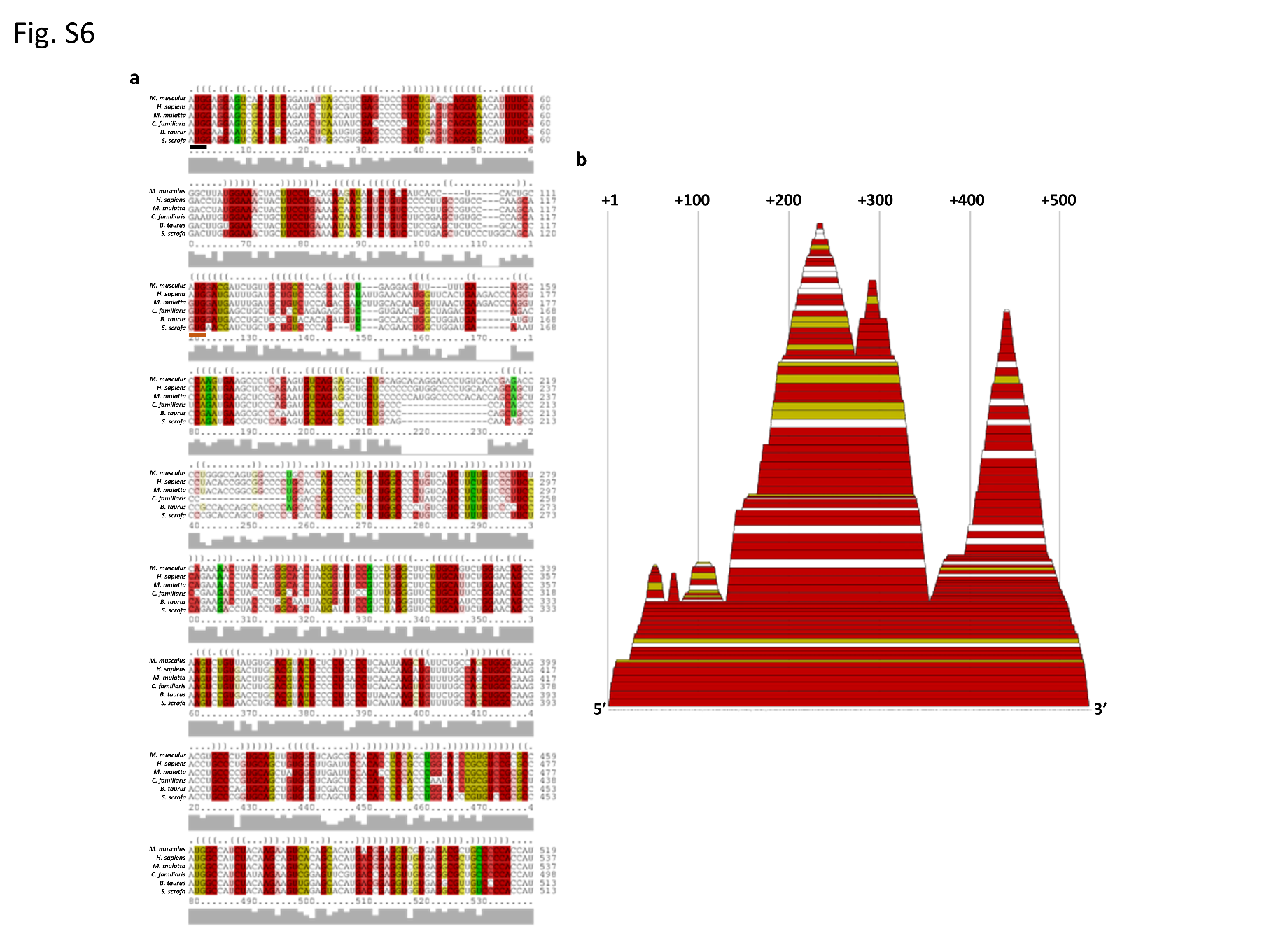


**Fig. S6. a)** Consensus secondary structure prediction of the aligned *p53* mRNA CDS from multiple species, conserved structures are indicated in red, positions of the 1^st^ and 2^nd^ AUG are indicated with lines. **b)** Mountain plot showing the consensus secondary structure of *p53* mRNA, plotted using the aligned *p53* mRNA sequences from multiple species. Peaks correspond to stem-loops, plateaus to loops, and slopes to helices. Base-pairing probabilities are indicated in different colors, red color indicates strong base-pairing probabbility and lighter colors indicate poor probabality of base-pairing. Consensus secondary structure prediction and mountain plot was generated using RNAalifold server in ViennaRNA web suite.


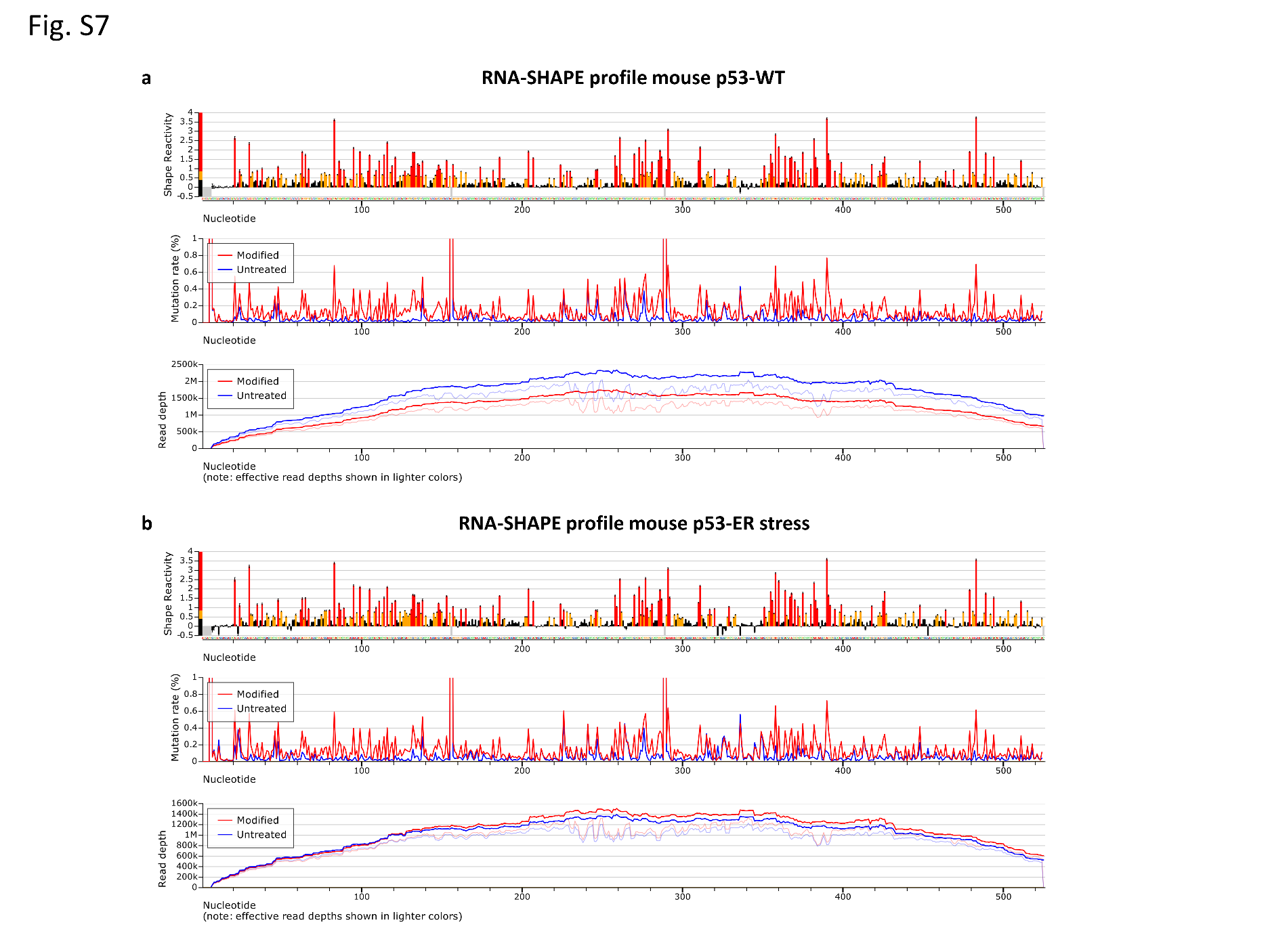


**Fig. S7.** RNA-SHAPE-MaP profiles of mouse *p53* mRNA under normal **(a)** and ER stress conditions **(b)**. The upper panel shows the SHAPE reactivity profiles, higher reactivities are indicated by red bars; the middle panel shows the mutation rate; and the lower panel shows the sequence read depth**.** RNA modified with 1M7 (modified) is indicated in red, and RNA treated with DMSO control (untreated) is indicated in blue. Related to the main figure 3.


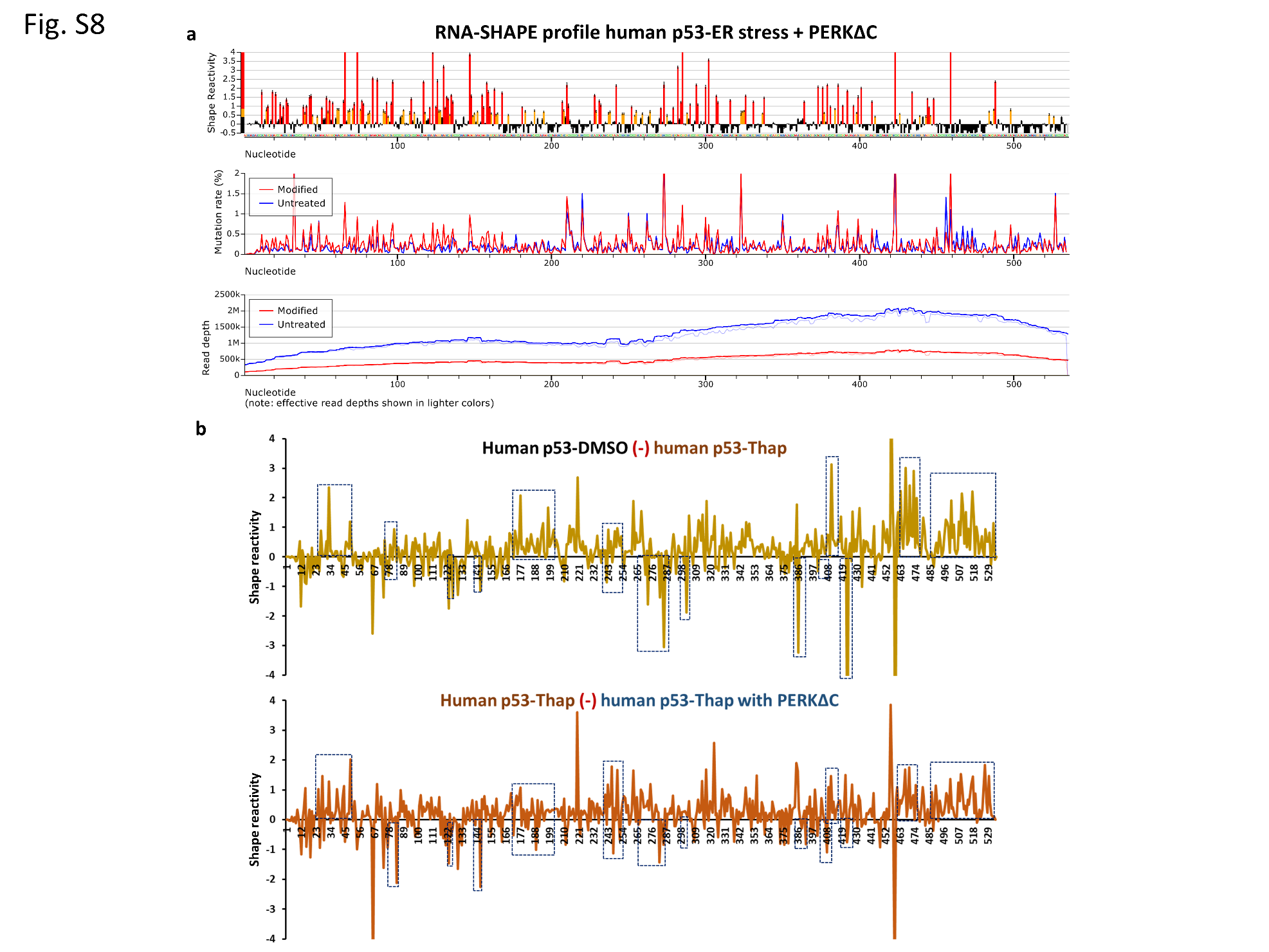


**Fig. S8. a)** RNA-SHAPE-MaP profile of human *p53* mRNA under ER stress conditions with PERKΔC overexpression. The upper panel shows the SHAPE reactivity profiles, higher reactivities are indicated by red bars; the middle panel shows the mutation rate; and the lower panel shows the sequence read depth**.** RNA modified with 1M7 (modified) is indicated in red, and RNA treated with DMSO control (untreated) is indicated in blue. **b)** Graphs showing the SHAPE reactivity differences of human *p53* mRNA, the upper panel shows SHAPE reactivity difference between normal (DMSO) and ER stress (Thap) conditions and the lower panel shows the reactivity difference between ER stress and ER stress with PERKΔC overexpression. ER stress induced reactivity differences that are reversed by PERKΔC are marked with dashed boxes. Related to the main figure 4c.


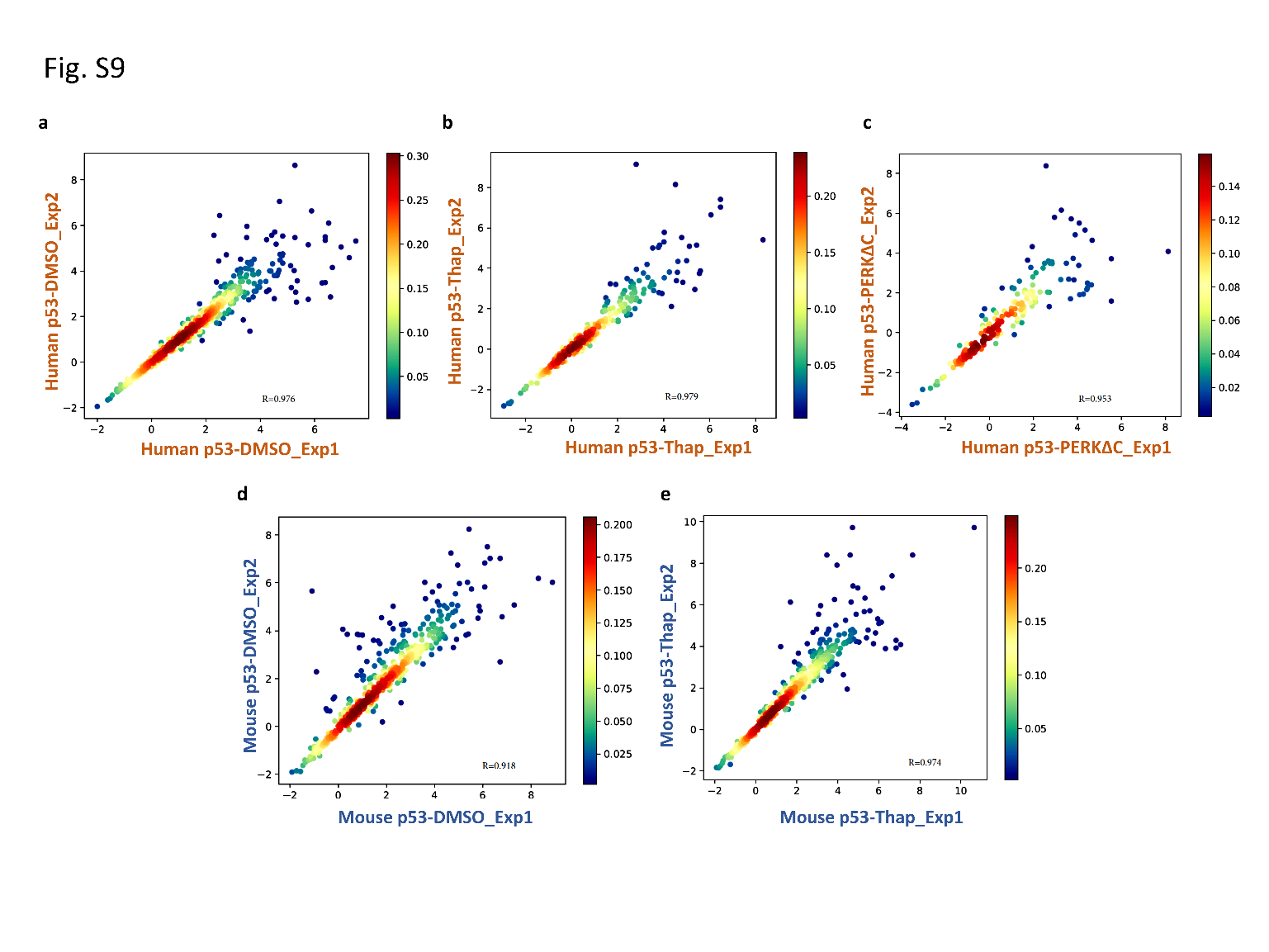


**Fig. S9.** Correlation analysis of RNA SHAPE-MaP from two biological replicates of the indicated experiment sets a) Human p53-DMSO; b) human p53-Thap; c) human p53-Thap+PERKΔC; d) mouse p53-DMSO; e) mouse p53-Thap. The biological replicates of each set of SHAPE-MaP experiment show excellent agreement with Spearman R value >0.9, indicating the reproducibility of the method. Related to main figures 2-4.


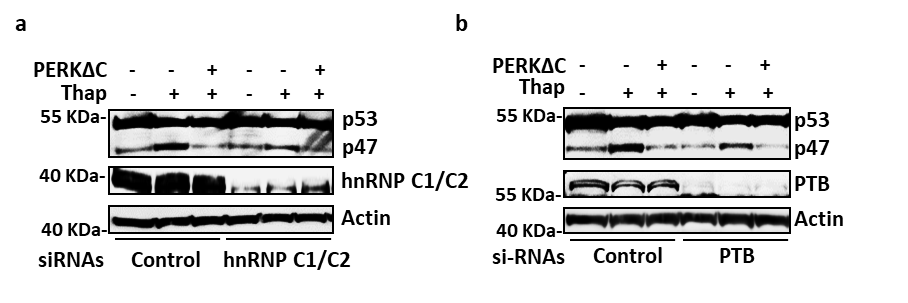


**Fig. S10.** Western blots showing the expression of p53 and p47, with cells knock-down with IRES transacting factors (ITAFs) hnRNP C1/C2 **(a)** and PTB **(b)** under normal and ER stress conditions. Knock-down of ITAFs has limited effects on the PERK-mediated synthesis of the p47 isoform during ER stress. Related to the main figure 4.


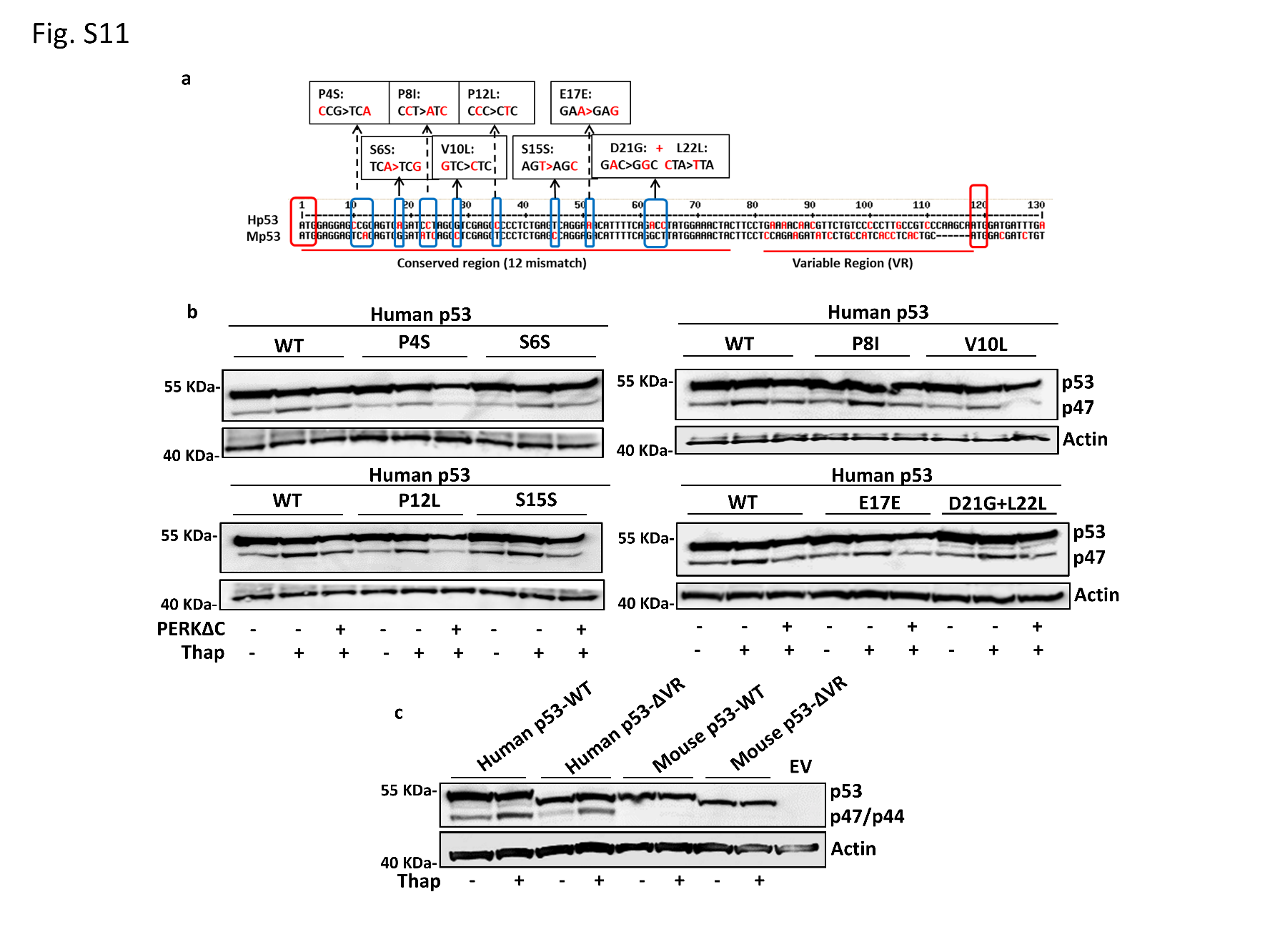


**Fig. S11.** Exchanging human p53 codons with the corresponding murine codons in the conserved region, or deletion of the variable region in the +1 to +118 region, does not affect p47 expression following ER stress. **a)** Illustration of the human and murine sequence between the first and second AUGs. The variable region located at +83 to the second AUG and the sequence differences between human and murine in the conserved region are illustrated. The boxes above show the murine mutations that were inserted into the human message. **b)** Expression of indicated constructs in human H1299 cells shows that changes in the conserved region of the human message do not affect the induction of p47 following Thap treatment. **c)** Deletion of the variable region does not affect induction of p47 under ER stress conditions. EV-vector control. Related to the main figure 5.

**Supplementary Table. 1: Oligos used for p53 RNA-SHAPE amplicon library preparation**

| **Serial no.** | **Name** | **Seqeunce (5’-> 3’)** |
| --- | --- | --- |
| 1 | RT primer human p53 | TCCACTCGGATAAGATGCT |
| 2 | Human p53_forward | ATGGAGGAGCCGCAGTCAGAT |
| 3 | Human p53_reverse | TCCACTCGGATAAGATGCT |
| 4 | RT primer mouse p53 | TTCCACCCGGATAAGATGCT |
| 5 | Mouse p53_forward | ATGACTGCCATGGAGGAGTCA |
| 6 | Mouse p53_reverse | TTCCACCCGGATAAGATGCT |
