# Supplementary material for: The p53 endoplasmic reticulum stress-response pathway evolved in humans but not in mice via PERK-regulated p53 mRNA structures
Source: Cell Death Differ. 2023 Feb 22;30(4):1072–81. doi: 10.1038/s41418-023-01127-y (PMC10070458; doi:10.1038/s41418-023-01127-y)

Original Western blots: cropped regions used in the figures are marked with dashed boxes.


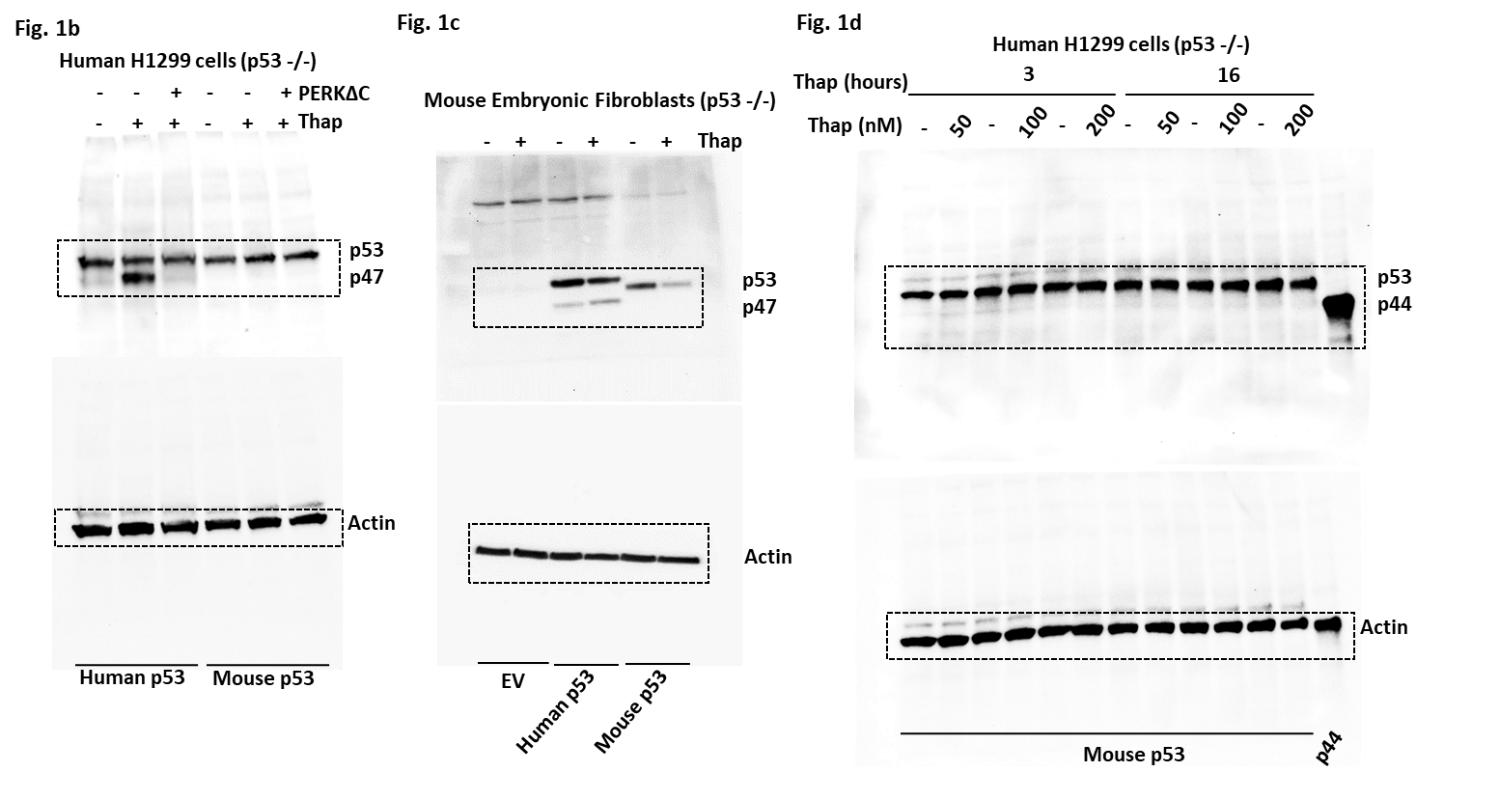


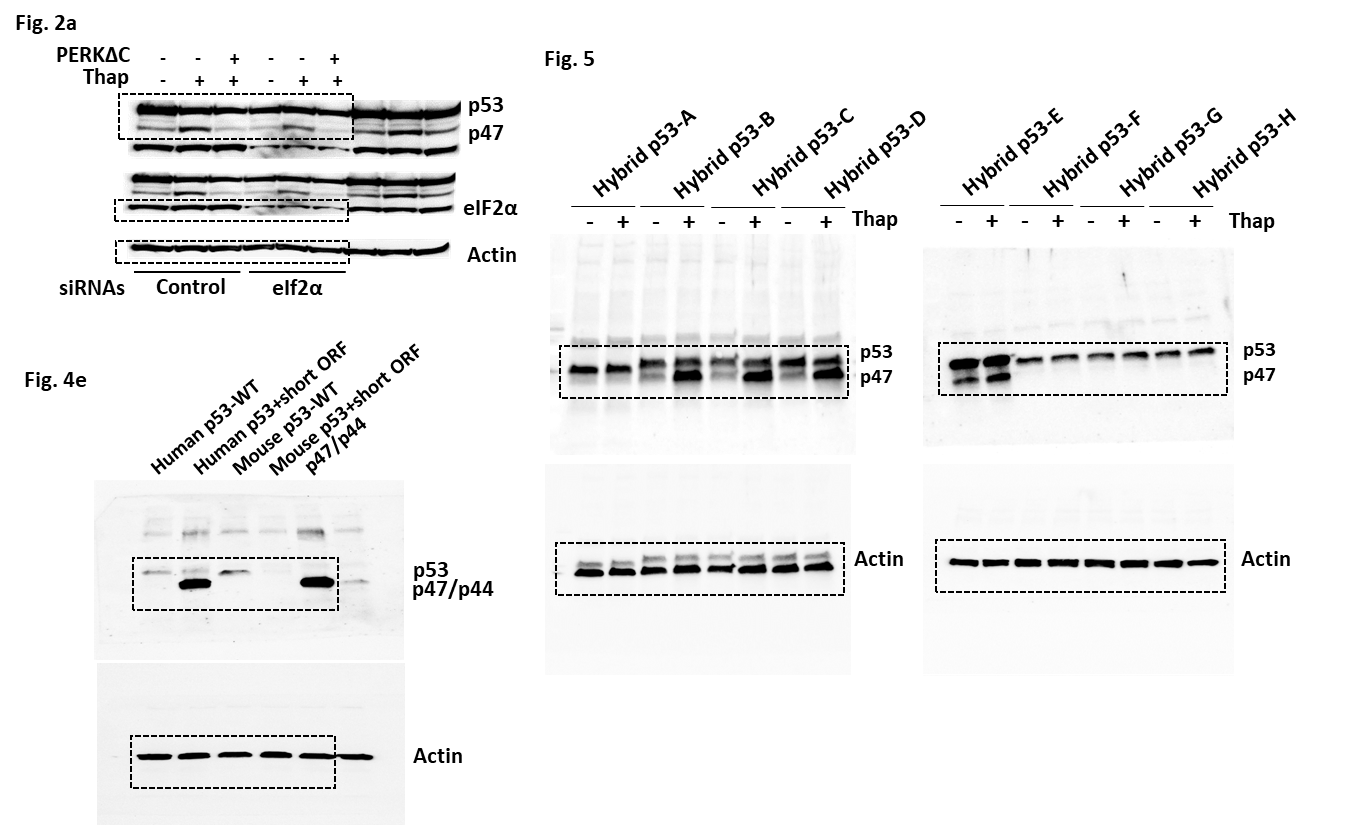


Original Western blots: cropped regions used in the figures are marked with dashed boxes.


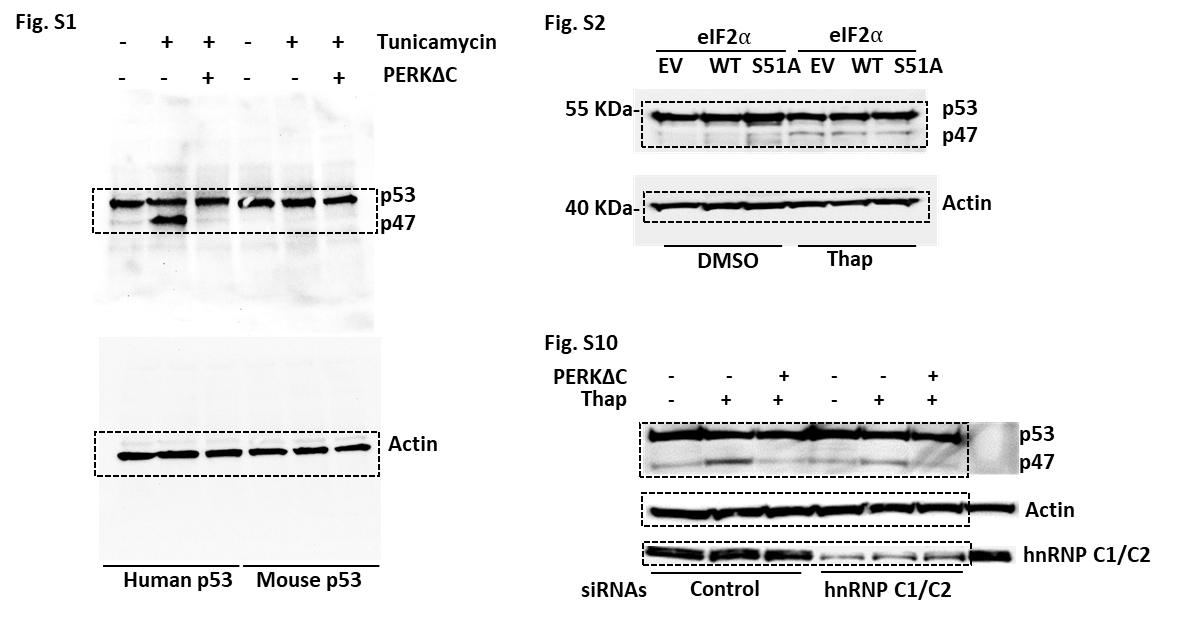


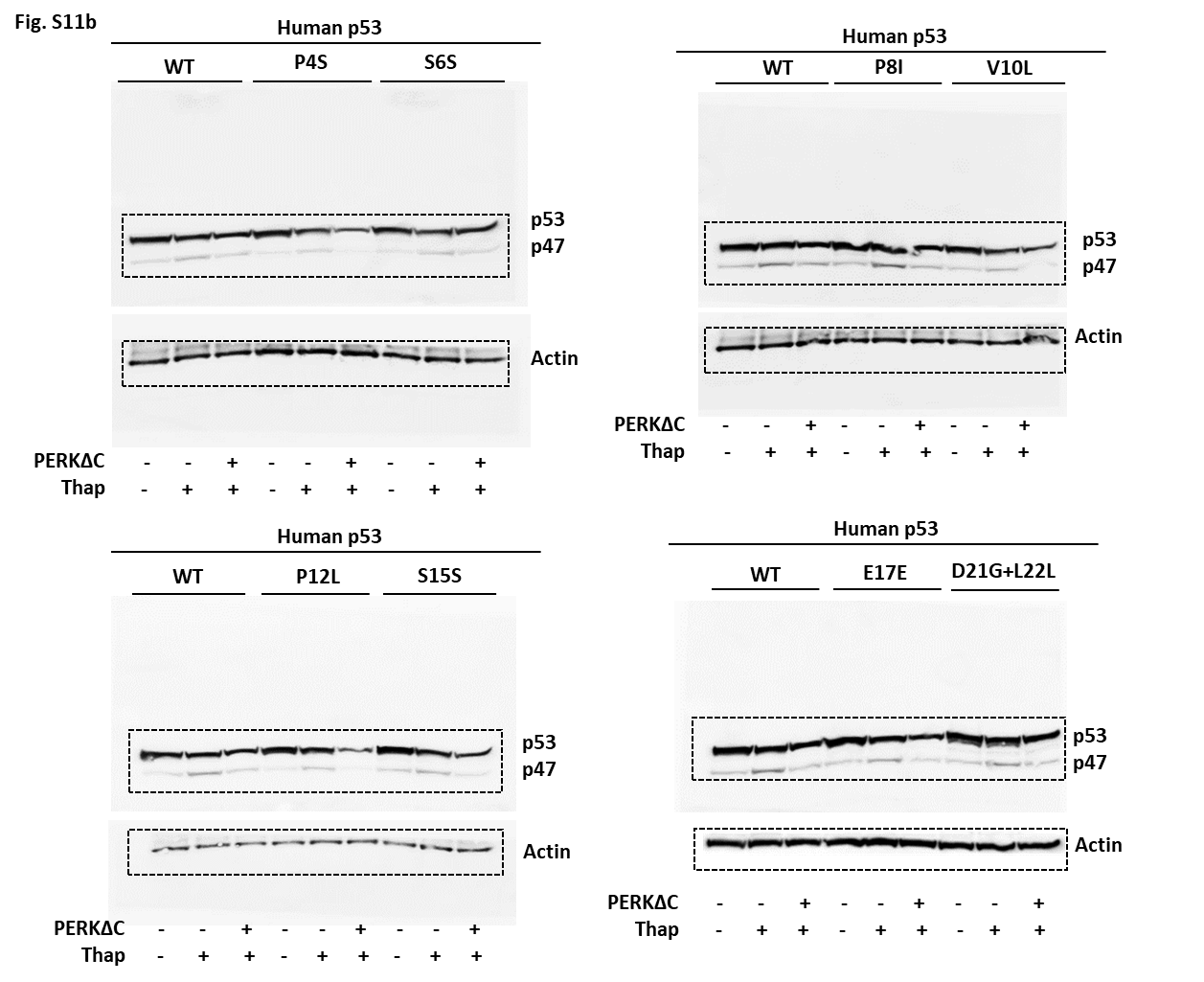

Supplement: Supplementary file 2 — Supplemental material_original Western blots [file 41418_2023_1127_MOESM2_ESM.docx]
